# The Roles of Thrombospondins in Hemorrhagic Stroke

**DOI:** 10.1155/2017/8403184

**Published:** 2017-10-30

**Authors:** Xuan Wu, Xu Luo, Qiquan Zhu, Jie Zhang, Yun Liu, Hansheng Luo, Yuan Cheng, Zongyi Xie

**Affiliations:** Department of Neurosurgery, The Second Affiliated Hospital, Chongqing Medical University, Chongqing 400010, China

## Abstract

Hemorrhagic stroke is a devastating cerebrovascular disease with significant morbidity and mortality worldwide. Thrombospondins (TSPs), as matricellular proteins, belong to the TSP family which is comprised of five members. All TSPs modulate a variety of cellular functions by binding to various receptors. Recently, TSPs gained attention in the area of hemorrhagic stroke, especially TSP-1. TSP-1 participates in angiogenesis, the inflammatory response, apoptosis, and fibrosis after hemorrhagic stroke through binding to various molecules including but not limited to CD36, CD47, and TGF-*β*. In this review, we will discuss the roles of TSPs in hemorrhagic stroke and focus primarily on TSP-1.

## 1. Introduction

Stroke, a major health issue globally, is the second leading cause of death worldwide and the main cause of adult disability [1]. Stroke is divided into two types, according to its etiological mechanisms. Ischemic stroke accounts for the majority of strokes, while hemorrhagic stroke is deadlier and poses a serious public health threat [2]. Intracerebral hemorrhage (ICH) and subarachnoid hemorrhage (SAH), two types of hemorrhagic stroke, are associated with significant morbidity and mortality globally [3–5]. However, the mechanism of brain injury after ICH and SAH is still not fully understood.

The thrombospondin (TSP) family contains five members divided into two subgroups. TSP-1 and TSP-2 compose subgroup A, while TSP-3, TSP-4, and TSP-5 belong to subgroup B. They were shown to regulate cell-cell and cell-matrix interactions by binding to various membrane receptors, other extracellular matrix proteins, and cytokines [6]. In recent years, the research and investigation on TSPs has increased due to their ability to participate in an extensive range of physiological and pathological processes including synaptogenesis, angiogenesis, apoptosis, platelet aggregation, inflammatory response, and wound repair [7, 8].

TSP-1–TSP4 are expressed in the brain [9–11]. They are implicated in synaptogenesis, angiogenesis, and inflammation as well as hydrocephalus after central nervous system (CNS) diseases which includes hemorrhagic stroke [12–23].

## 2. Methods

TSPs have been getting increased attention in the area of hemorrhagic stroke research. Our literature review indicated that TSPs play diverse roles in hemorrhagic stroke. To better understand their roles in hemorrhagic stroke, the literature search focused mainly on TSP receptors, as there are many. In addition, to elucidate the underlying molecular mechanisms of TSPs in hemorrhagic stroke, we also searched literature regarding TSPs in other diseases, particularly CNS diseases.* PubMed, Web of Science, *and* SwetsWise *were the databases utilized in our literature review. After carefully reading the articles, we summarized the roles of TSPs in hemorrhagic stroke and discussed the relevant mechanisms.

### 2.1. TSP Family

**Figure 1:**
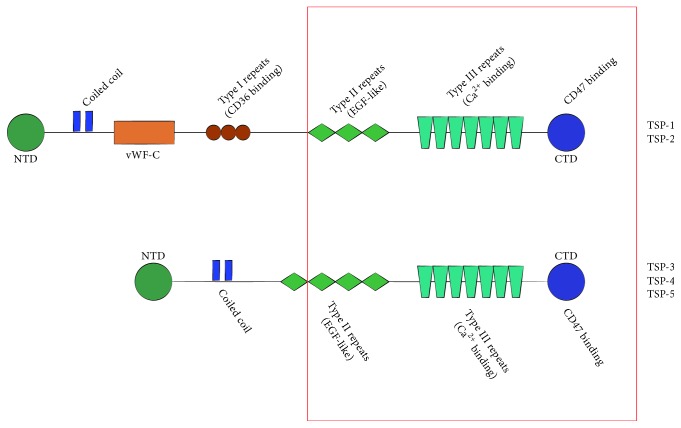
Schematic representation of TSPs. All of TSPs share highly homologous CTD, Type 2 repeats, and Type 3 repeats (red region), while TSP-1 and TSP-2 have vWF-C domain and Type 1 repeats. NTD may be characteristic to the family members. TSPs have a complex multidomain architecture that provides an option to bind various ligands. For instance, CTD is involved in CD47 binding, while Type III repeats contain Ca^2+^ binding site. Type I repeats are implicated in interaction with CD36, a receptor for TSP1 and TSP2, and inhibition of MMPs, while vWF-C is responsible for binding members of the TGF-*β* superfamily. This figure is only a partial listing. CTD: C-terminal domain, EGF-like: epidermal growth factor-like, vWF-C: von Willebrand factor *C*-type, and NTD: N-terminal domain.

All TSPs are highly homologous at the protein level, especially in their “signature domain,” C-terminal domain, followed by calcium-binding domains, and thirteen EGF-like Type 3 repeats [24]. TSPs are divided into two subgroups due to their disparate N-terminal domains [25] (Figure 1). It is worth mentioning that the transforming growth factor-*β* (TGF-*β*) binds to TSP-1 through the vWF-C. Thus, the subgroup B of TSP cannot activate latent TGF-*β*. The TSP family participates in various physiological and pathological processes through these different functional domains.

TSP-1, one of the members of TSP subgroup A, is a 420–450 kDa trimeric glycoprotein [26, 27]. As a matricellular protein, it was initially isolated from human blood platelets [28, 29]. A variety of cells can produce TSP-1, including endothelial cells (ECs), monocytes/macrophages, smooth-muscle cells, and leukocytes [30–33]. In addition, activated astrocytes can secrete TPS-1 [34, 35]. Certainly, the main sources of TSP-1 are platelets [28]. As a multifunctional protein, TSP-1 has diverse biological roles by interacting with various proteins, cell surface receptors, and proteoglycans of specific domains [36–38] (Table 1).

TSP-2, another member of TSP subgroup A, is similar in structure to TSP-1. It is involved in angiogenesis, synaptogenesis, regulating the cell-matrix interaction, and protecting the integrity of matrix [17, 39]. Some critical differences between TSP-1 and TSP-2 were reported. TSP-1 and TSP-2 were detected at different time points and had different peaks in a wound healing assay [40]. Moreover, the different expression of TSP-1 mRNA and TSP-2 mRNA were at disparate time points after ICH [17]. Thus TSP-1 and TSP-2 may exert diverse effects in different stages during ICH.

TSP-3, TSP-4, and TSP-5, belonging to TSP subgroup B, are structurally different from TSP subgroup A (Figure 1). Recently, multiple properties of TSP-4 have been demonstrated, such as having an angiogenic effect and regulating vascular inflammation, extracellular matrix (ECM) remodeling, skin wound healing, and atherosclerotic lesions [41, 42].

### 2.2. Role of TSPs in Angiogenesis after ICH

As the source of oxygen and glucose for the brain is supplied by the blood, angiogenesis is pivotal for growth and repair of brain [13]. The process of angiogenesis is due to the effect of various proangiogenic and antiangiogenic factors [43]. Antiangiogenic factors are critical for forming new vasculature by inhibiting excessive growth [44]. Wang et al. found an elevated vascular density during retinal vascular development and less susceptibility to hypoxia-mediated disruption of blood vessels in TSP-1 null mice [45]. Furthermore, Bornstein et al. discovered obvious disarrangement of the ECM and denser vascular density during wound repair in TSP-2 null mice [39]. These findings indicate that TSP-1 and TSP-2 are antiangiogenic factors. Subsequently, some researchers found that the expression of TSP-1 and TSP-2 was increased in the process of angiogenesis after ICH [46, 47]. It suggests that TSP-1 and TSP-2 are involved in angiogenesis following ICH.

**Figure 2:**
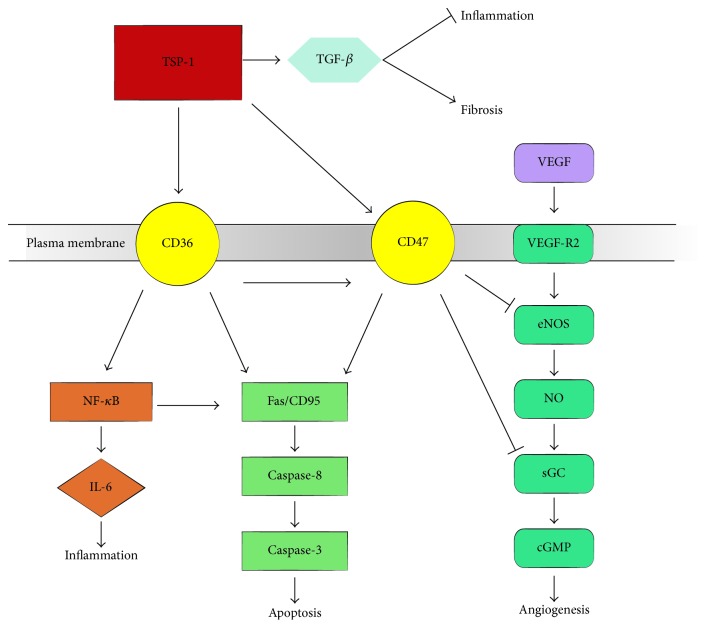
The roles of TSP-1 in diverse pathological processes. TSP-1 interacting with CD47 or CD36 suppressed VEGF-induced angiogenesis to inhibit NO signaling pathway. Moreover, TSP-1 binding to CD36 or CD47 induced apoptosis. TSP-1 exerted proinflammatory effect by elevating the levels of IL-6. However, TSP-1 played an anti-inflammatory role by regulating TGF-*β* and inhibiting NO-mediated vascular cell responses. In addition, TSP-1 mediated fibrosis through a TGF-*β* signaling pathway.

To explore the role of TSP-1 and TSP-2 in angiogenesis after ICH, Zhou et al. investigated the expression of TSP-1 and TSP-2 after ICH [13]. Abundant TSP-1- and TSP-2-immunoreactive microvessels were observed in the perihematomal region and then extended into the clot after ICH in rat models. Poor immunoreactivity in microvessels was detected in sham-operated group. It suggests that TSP-1 and TSP-2 could inhibit angiogenesis after ICH. In addition, TSP-1 could hinder the vascular endothelial growth factor- (VEGF-) induced angiogenesis via inhibition of NO signaling by interacting with CD47 or CD36. The alteration of TSP-1 might present a negative-feedback mechanism in angiogenesis in hemorrhagic brains [48, 49, 57] (Figure 2).

Paradoxically, there are also evidences that TSP-1 and TSP-2 can promote angiogenesis. In 1994, Nicosia and Tuszynski observed that TSP-1 promoted the formation of microvessels* in vitro* [58]. However, the mechanisms were not characterized. Subsequently, Qian et al. measured the capacity of bovine aortic EC to invade and form microvessel-like tubes in a collagen gel. They showed that TSP-1 can increase EC tube formation at low concentrations but inhibited EC tube formation at higher concentrations [55]. This biphasic effect was related to the stimulation of matrix metalloproteinase-9 (MMP-9) activity by TSP-1. Nevertheless, these results were restricted in that bovine aortic EC and not microvascular EC was used in the experiments. Additional evidence for an angiogenic function of TSP-1 and TSP-2 was found by Yang and colleagues. Yang et al. investigated whether thrombin, a proangiogenic factor, could mediate the expression of TSP-1 and TSP-2 in the brain of ICH rats [17]. The result showed that the expression of TSP-1 and TSP-2 was dramatically decreased after administration of hirudin, a specific thrombin inhibitor, compared with sham-operated animals. On the other hand, intracerebral injection of thrombin markedly increased the expression of TSP-1 and TSP-2. It is rational to speculate that TSP-1 and TSP-2 may promote an angiogenic function by suppressing ECs migration and promoting ECs apoptosis, which leads to the development of new lumen and the maturation of vascular structures [59, 60].

However, the expression of TSP-1 mRNA and TSP-2 mRNA were at diverse time points after ICH [17]. Zhou et al. observed that an obvious increase in TSP-1 mRNA during the early period of ICH might be associated with its inhibitory effect on antiangiogenesis [13]. In contrast, the upregulation of TSP-2 during the later stage following ICH might facilitate the stability of newly formed blood vessels by reducing the level of MMPs and ultimately contribute to the less degradation of the ECM [17, 50, 56]. Taken together, TSP-1 and TSP-2 may execute different effects in angiogenesis after ICH.

In general, these findings indicate that TSPs play a significant role in the process of angiogenesis after ICH. Both TSP-1 and TSP-2 expression were increased after ICH. Previous studies showed that TSP-1 and TSP-2 not only play an antiangiogenic role but also promote angiogenesis. We speculate that the biphasic effect of TSP-1 and TSP-2 may be concentration-related and time-dependent.

### 2.3. Role of TSP-1 in Inflammation after ICH

After ICH, blood components immediately enter the intracerebral spaces and induce an inflammatory reaction [61]. The inflammatory reaction is involved in depletion of dead cell and other residues and activation of repair signals, a significant defense reaction to brain injury after ICH. A prolonged inflammatory reaction could contribute to detrimental reconstruction [62].

TSP-1 could enhance the release of IL-6 from macrophages by interacting with CD36 in rat myocardial infarction model [51]. NF-*κ*B can activate interleukin-6 (IL-6), which have crucial role in regulating the immune response by prompting the differentiation of B lymphocyte [63]. Interestingly, the activation of NF-*κ*B is decreased in macrophages of patients with CD36 deficiency [64]. These results suggest that TSP-1 may induce inflammatory response through NF-*κ*B pathway. Additional support for a proinflammatory function of TSP-1 has been contributed by Xing and coworkers. Xing et al. showed that exposure of human brain ECs to low concentration of TSP-1 caused a prompt and obvious upregulation in inflammatory adhesion molecules, such as intercellular adhesion molecule-1 (ICAM-1) and vascular cell adhesion molecule-1 (VCAM-1). This function is done by binding CD47 receptor [15].

Although a lot of experimental studies demonstrate a proinflammatory function of TSP-1, increasing evidence supports an anti-inflammatory function of TSP-1. TSP-1 is a prime activator of TGF-*β*, which are anti-inflammatory cytokines [65]. Cekanaviciute et al. showed that suppressing astrocytic TGF-*β* signal contributed to stronger and broader inflammatory response in the peri-infarct cortex after ischemic stroke in mouse model [53]. Furthermore, Yang et al. showed that the expression of TSP-1 was increased at the early phase after ICH, and the upregulation of TSP-1 expression was associated with its inhibitory effect on inflammatory responses after brain injury [17]. The mechanism underlying anti-inflammatory function of TSP-1 may be related to inhibiting NO-mediated vascular cell responses by binding to CD47 or CD36 [45].

In brief, TSP-1 may inhibit the inflammatory response by activating TGF-*β* or through the NO-mediated vascular cell responses, whereas it may induce inflammatory response through NF-*κ*B pathway following ICH. It is supposed that the double effects of TSP-1 on inflammatory response are time-dependent. The exact mechanism of TSP-1 on the inflammatory response requires further research.

### 2.4. Role of TSP-1 in Apoptosis after SAH

Apoptosis is a process of programmed cell death and can also be induced by multiple pathological stresses. The first time TSP-1 was directly connected to apoptosis was in a cancer study in 1997 [66].

TSP-1 promoted apoptosis in ECs by binding to CD36, which could partially induce cerebrovasospasm after SAH [14]. CD47, another receptor of TSP-1, can promote Fas/CD95-mediated apoptosis in ECs and neutrophils [67, 68]. Another study demonstrated that TSP-1-induced apoptosis in brain microvascular endothelial cells could be regulated by TNF-R1 (tumor necrosis factor receptor 1) [20]. In addition, some evidences showed that the reaction of TSP-1 and CD47 plays a role in neuronal death or survival. Exposure of cultured cerebral cortical neurons to TSP (0.5–10 *μ*g/mL) for 24 h induced a dose-dependent cell death. Pretreatment with a CD47 blocking antibody for 1h markedly decreased TSP-induced neuronal death through caspase-3-dependent and caspase-independent pathways [18, 19]. It is reasonable to speculate that TSP-1 plays a role in the process of apoptosis by binding to CD36 or CD47 during SAH.

### 2.5. Role of TSP-1 in Fibrosis after SAH

SAH not only contributes to vasospasm but also causes subarachnoid fibrosis [69]. Compelling evidences demonstrated that fibrosis of the arachnoid granulations and leptomeninges might contribute to the progress of posthemorrhagic communicating hydrocephalus by decreasing the drainage of cerebrospinal fluid (CSF), hindering the flow of CSF, and reducing CSF absorption [16]. Antifibrinolytic therapy, widely used for reducing the rate of rebleeding in patients with SAH, has not been shown to be associated with the development of hydrocephalus or delayed brain injury after SAH [70].

TSP-1 binds to the small latent complex consisting of the N-terminal prodomain, known as the latency associated peptide (LAP), and the C-terminal portion of the latent complex, known as mature TGF-*β* [7]. The leucine-serine-lysine-leucine (LKSL) at the amino terminus of LAP that is significant for LAP to interact with the KRFK sequence of TSP-1 and the modulation of latent TGF-*β* activation by TSP-1 [54]. In addition, the LKSL peptide could reduce hepatic fibrosis and renal interstitial fibrosis [52, 71, 72]. To determine whether LKSL could protect against subarachnoid fibrosis, Liao et al. investigated the role of LKSL in subarachnoid fibrosis after SAH [21]. Their results revealed that LKSL treatment alleviated subarachnoid fibrosis, delayed the progress of chronic hydrocephalus, and prevented ventriculomegaly formation by suppressing TSP-1-mediated TGF-*β* signaling pathway. These findings suggest that TSP-1 may serve as a promising target for future therapeutic strategy of subarachnoid fibrosis following SAH.

### 2.6. Role of TSPs in Synaptogenesis in Other CNS Diseases

Synaptogenesis is important for motor function recovery after various CNS injuries. Increasing evidences suggest that astrocytes are critical in the formation of synapses [73]. TSPs are responsible for the ability of astrocytes to enhance synaptic development* in vitro *[12].

Christopherson et al. observed that both TSP-1 and TSP-2, secreted by astrocytes, could increase CNS synaptogenesis in rat retinal ganglion cells (RGCs) [12]. Moreover, Xu et al. found that TSP-1 promoted synaptogenesis in the early phase of neuronal development in cultured rat hippocampal neurons, but it failed to induce synaptogenesis in mature neurons [22]. This synaptogenic effect of TSP-1 is mediated by neuroligin 1, a membrane protein involved in the formation of CNS synapses [22]. In addition, treatment with TSP-1 increased excitatory synaptogenesis in astrocyte-null hippocampal neuronal cultures [24]. In addition, TSP-4 was expressed in astrocytes, cerebrovascular smooth-muscle cells and ECs and was implicated in adhesion of retinal ganglion cells and axonal outgrowth in the developing retina [74–76].

All TSPs exerted synaptogenic effects by their EGF-like domains directly binding with *α*2*δ*-1, which were rich in substantial neurons [77, 78]. Overexpression of *α*2*δ*-1 in neurons increased the number of synapses* in vivo* [77]. However, the role of TSPs in synaptogenesis after hemorrhagic stroke need to be further studied.

### 2.7. Role of TSP-1 in Evaluation of Severity and Prognosis after Hemorrhagic Stroke

#### 2.7.1. Plasma TSP-1 Concentration

TSP-1 expression was increased after traumatic, ischemic, and hemorrhagic brain injuries in animal cortex [17, 79, 80]. To evaluate the relationship between plasma concentrations of TSP-1 and the severity of hemorrhagic stroke, researchers assessed the levels of TSP-1 in peripheral blood. The results showed that plasma TSP-1 concentration was tightly related to the severity and 6-month clinical outcome following SAH [81]. Additionally, TSP-1 was considered as an independent predictor of 1-week mortality, 6-month mortality, 6-month total survival, and 6-month poor prognosis after ICH [82]. Its predictive value was similar to NIHSS score and hematoma volume under ROC curves [82]. The plasma TSP-1 is thought to be released from circulating blood cells or from the CNS. However, the correlation between plasma TSP-1 levels and platelet count remains unclear [81, 82].

#### 2.7.2. CSF TSP-1 Concentration

Cerebrospinal fluid (CSF) TSP-1 concentration was also altered after hemorrhagic stroke. Chen et al. explored the correlation between CSF TSP-1 concentration and the severity of hemorrhagic stroke [14]. They reported that CSF TSP-1 levels were significantly elevated and reached peak on days 1–3. In addition, others discovered that patients with unfavorable prognosis or vasospasm had higher CSF TSP-1 concentration on days 1–3 and days 5–7 after SAH than those with favorable prognosis or without vasospasm [14]. The increased TSP-1 may be released from leukocytes, platelets in the bloody CSF, or the ECs of the ruptured blood-brain barrier (BBB) and even from the impaired brain tissue [35]. It is believed that TSP-1 may be a potential prognostic biomarker of hemorrhagic stroke.

## 3. Conclusions and Perspectives

TSPs, as multifunctional proteins, exert diverse functions by binding with various receptors through their distinct domains. TSP-1 participates in angiogenesis, the inflammatory response, apoptosis, and fibrosis following hemorrhagic stroke. Moreover, increased concentration of TPS-1 in both plasma and CSF indicates poor prognosis after hemorrhagic stroke. Future studies are needed to further determine the cellular and molecular mechanisms by which TSP-1 contributes to hemorrhagic stroke. This may lead to the identification of new therapeutic targets.

## Figures and Tables

**Table 1:** Diverse roles of TSP-1.

Binding receptors or molecules	Functions	References
CD36	Antiangiogenic	[13, 45–50]
	Proinflammatory	[15, 51]
	Anti-inflammatory	[17, 45]
	Proapoptotic	[14]
	Profibrogenic	[21, 52]

CD47	Antiangiogenic	[49]
	Anti-inflammatory	[45]
	Proapoptotic	[18, 19]

TNF-R1	Proapoptotic	[20]

TGF-*β*	Anti-inflammatory	[53]
	Profibrogenic	[54]

MMP-9	Antiangiogenic	[55, 56]

*α*2*δ*-1, neuroligin 1	Prosynaptogenic	[22, 24]
